# Eunuchs Do Not Take the Gout

**Published:** 1956-10

**Authors:** G. D. Kersley


### "EUNUCHS DO NOT TAKE THE GOUT"

BY

G. D. KERSLEY, T.D., M.D., F.R.C.P.

Throughout the ages gout has always been linked with sex and drink. The disease is certainly becoming rare—are we not the men we were?

Gout has been described by Warner as the "dominus morborum et morbus dominorum"—the disease of the erudite and the greatest of all diseases.

Williams Cadogan commences his fifteenth-century essay on gout with the remark that "to enjoy good health is better than to command the world". He then goes on to give the background of gout, "If I am not mistaken the laudible qualities, which are at present the most in fashion, are keeping mistresses, debauching friends' wives, cheating at the gaming tables and at Newmarket, indulging in every excess and refinement in eating and drinking and speaking in Parliament."

Garrod, two hundred years later, dates gout back to antiquity, from the time when man first indulged in luxury.

The link with learning and leadership has long been recognized and may be typified by Harvey, Linné, Widal, Newton, Leibniz, Wallerstein, the two Pitts and Fox. Sir Thomas Sydenham, sometimes described as the modern Hippocrates of Medicine, writing in the seventeenth century, remarks that "gout rarely attacks fools", but adds modestly "those who chose may except the present writer". With regard to alcohol, he was lenient—"drink wine, have the gout—drink no wine and have the gout" and adds "I count water by itself crude and injurious". In those days he was certainly right.

Gout has always been considered a difficult disease to cure and there is an old Spanish proverb "with aspect to the gout, the Physician is but a lout". But did not Rousseau proclaim the need for "more perfect physics, more honest physicians, more enlightened statesmen and wiser mankind"? Lucien wrote a burlesque tragedy on the powers of the Goddess Podegra and the inefficiency of treatment.

Some of the treatments were fantastic—Walpole recommended the wearing of the "Bootikins" and goes on to say "the physicians and apothecaries who began by recommending them, now finding them specific cry them down". Yet a Bishop of Chichester was sanctified as St. Richard for lending his boots to be placed on the feet of the poor to cure the gout.

Horace Walpole had little use for Spas: "Bath is excellent for those in travail for the gout, but I certainly have no occasion to accelerate the attacks." This raises the question of precipitation of an attack by hydrotherapy which was often advised by the older physicians, even twenty years ago, as being most laudible. I never knew whether this was really to avoid the wrath of a patient who developed an acute attack during treatment.

Goethe states that to beguile the tedium of spa treatment he always engaged in some new love affair, but we know now that the excitement of stress of coitus will provoke an attack of gout.

The connexion of sex and gout certainly dates back to antiquity. Hippocrates wrote: "Eunuchs do not take gout or become bald, a woman does not take gout unless her menses be stopped, a young man does not take gout unless he indulges in coition."

Walpole, however, adopts the attitude "its an ill wind which blows no one good". "Since I must grow old and have the gout, I have long turned those disadvantages to my account and plead them to the utmost when they will save me from anything I dislike."

The term gout seems to be derived from the word gutta used by Archimataeus in the twelfth century—"gutta or gout occurs in various parts of the body, but chiefly in the joints. . . . The disease is called gout because it is caused by rheumy humours that flow drop by drop to the parts mentioned."

Now let us turn to the classification of abarticular gout. Sir Richard Blackmore in 1735 divides gout in three ways (1) hereditary or contingent, (2) "perfect", when there is an acute attack with expulsion of the impure particles into the joints and tophi, or "imperfect" when impurities are left behind and travel to other organs and (3) simple or mixed when complicated by other conditions such as stone.

Professor Palmier in 1769 includes under gout in his *Traité methodique et dogmatique de la Goutte* syphilitic gout, tumours, contusions, haemorrhoids, menstrual irregularities and almost any other disease one can conceive.

Sir Archibald Garrod, in 1876, expresses the classification of his day—accepted until fairly recently by many into (1) regular gout, (2) retrocedent or metastatic gout, suddenly flying to the stomach, intestine, heart or head, (3) abarticular gout of the digestive tract, heart or respiratory, urinary or ophthalmic system, and (4) diseases to which gouty patients are prone—gravel and calculus, sciatica and lumbago, phlebitis, kidney disease, diabetes, plumbism and pyaemia. He, however, for the first time stresses the importance of excess uric acid in the blood and crystals in the tissues in diagnosis. He also describes very beautifully the histology of the kidneys in gout. Wollaston had, however, previously, in 1797, noted uric acid in tophi.

Scudamore, 1819, explains further the theory of retrocedent gout—"when during the existence of gouty inflammation in either its acute or chronic form, a sudden cessation of the external action takes place, it sometimes happens that an internal organ becomes immediately and violently affected. The most common symptoms are acute abdominal pains and sickness or enteritis or sometimes apoplexy. Danger is pressing and, unless relief be speedily rendered, death soon closes the scene".

Alternation of visceral symptoms in gout is frequently stressed. Walpole writes "a cough is vexatious, but in old persons is a great preservative. It is one of the forms in which gout appears, and exercises and clears the lungs." He goes on "if it is content to act the personage of a cough, pray humour it, it will prolong your life if you do not contradict it and fling it somewhere else." He also states that Lord Buckingham killed himself by curing the gout with a cold application to the foot and causing an apoplexy. Even the great Trousseau said in one of his lectures "Gentlemen, ought we to treat the gout? . . . When I see a patient in an acute attack I cross my arms and do nothing—absolutely nothing."

Alternation of attacks of gout and depression are noted in the younger Pitt and suggest a psychosomatic or allergic element. Renal calculus and gout have long been connected together. Erasmus writes: "Thou hast the gravel and I the gout—we have married two sisters."

This is a little of the folk-lore attached to gout but frequently in ancient saws lurk grains of wisdom.

### BIBLIOGRAPHY

Archimataeus of Salerno. Quoted Cohen, H. (1948) *Textbook of Rheumatic Diseases.* Copeman. London.
Bohn (1855). *Handbook of Proverbs*, 31.
Cadogan, W. "Essay on gout." Quoted (1925) *Ann. Med. Hist.*, 64.
Duckworth, Sir R. (1735). *Discourse on Gout, Rheumatism and the Kings Evil.*
Garrod, A. B. (1948). *Med. Chir. Trans. Lond.*, **31**, 83.
Goethe. Quoted (1932) *Brit. Med. J.*, **1**, 670.
Hippocrates. Translation Vol. 1 and 2. Sydenham. Soc. Lond. 1849.
Palmier, Prof. (1769). *Traité methodique et dogmatique de la Goutte.*
Scudamore, C. (1819). *Treatise on Gout.*
Sydenham, T. (1683). *Tactatus de Podagra*, London. Quoted (1925) *Ann. Med. Hist.*, 171.
Walpole, H. Quoted (1934) *St. Bart. Hosp. J.*, 41. Also letter to Sir Horace Mann, 1785.
Wollaston, W. H. (1797). *Phil. Trans. Lond.*, **87**, 386.

